# Challenges in characterization of transcriptomes of extracellular vesicles and non-vesicular extracellular RNA carriers

**DOI:** 10.3389/fmolb.2023.1327985

**Published:** 2023-12-05

**Authors:** Julia Makarova, Diana Maltseva, Alexander Tonevitsky

**Affiliations:** ^1^ Faculty of Biology and Biotechnology, HSE University, Moscow, Russia; ^2^ Shemyakin-Ovchinnikov Institute of Bioorganic Chemistry RAS, Moscow, Russia; ^3^ Hertsen Moscow Oncology Research Center, Moscow, Russia; ^4^ Art Photonics GmbH, Berlin, Germany

**Keywords:** extracellular vesicles, exosomes, miRNA, extracellular RNA, exomeres, supermeres

## Abstract

Since its original discovery over a decade ago, extracellular RNA (exRNA) has been found in all biological fluids. Furthermore, extracellular microRNA has been shown to be involved in communication between various cell types. Importantly, the exRNA is protected from RNases degradation by certain carriers including membrane vesicles and non-vesicular protein nanoparticles. Each type of carrier has its unique exRNA profile, which may vary depending on cell type and physiological conditions. To clarify putative mechanisms of intercellular communication mediated by exRNA, the RNA profile of each carrier has to be characterized. While current methods of biofluids fractionation are continuously improving, they fail to completely separate exRNA carriers. Likewise, most popular library preparation approaches for RNA sequencing do not allow obtaining exhaustive and unbiased data on exRNA transcriptome. In this mini review we discuss ongoing progress in the field of exRNA, with the focus on exRNA carriers, analyze the key methodological challenges and provide recommendations on how the latter could be overcome.

## 1 Introduction

Since its initial discovery in 2008, exRNA has been found in all human biological fluids including blood, saliva, milk, urine, bile, sweat, sputum, lacrimal, seminal, amniotic, cerebrospinal, synovial fluids, and ascites (Mateescu et al., 2022). In the following years, it became apparent that exRNA represents a complex mixture of RNase-stable RNA biotypes and their parts, including miRNA, tRNA, YRNA, snRNA, sno/scaRNA, vault RNA, piRNA, mRNA, lncRNA, rRNA, and circRNA (Dellar et al., 2022). To remain stable in RNase-rich cell-free milieu, exRNA must be protected by association with a certain carrier (Arroyo et al., 2011; Turchinovich et al., 2011).

**FIGURE 1:**
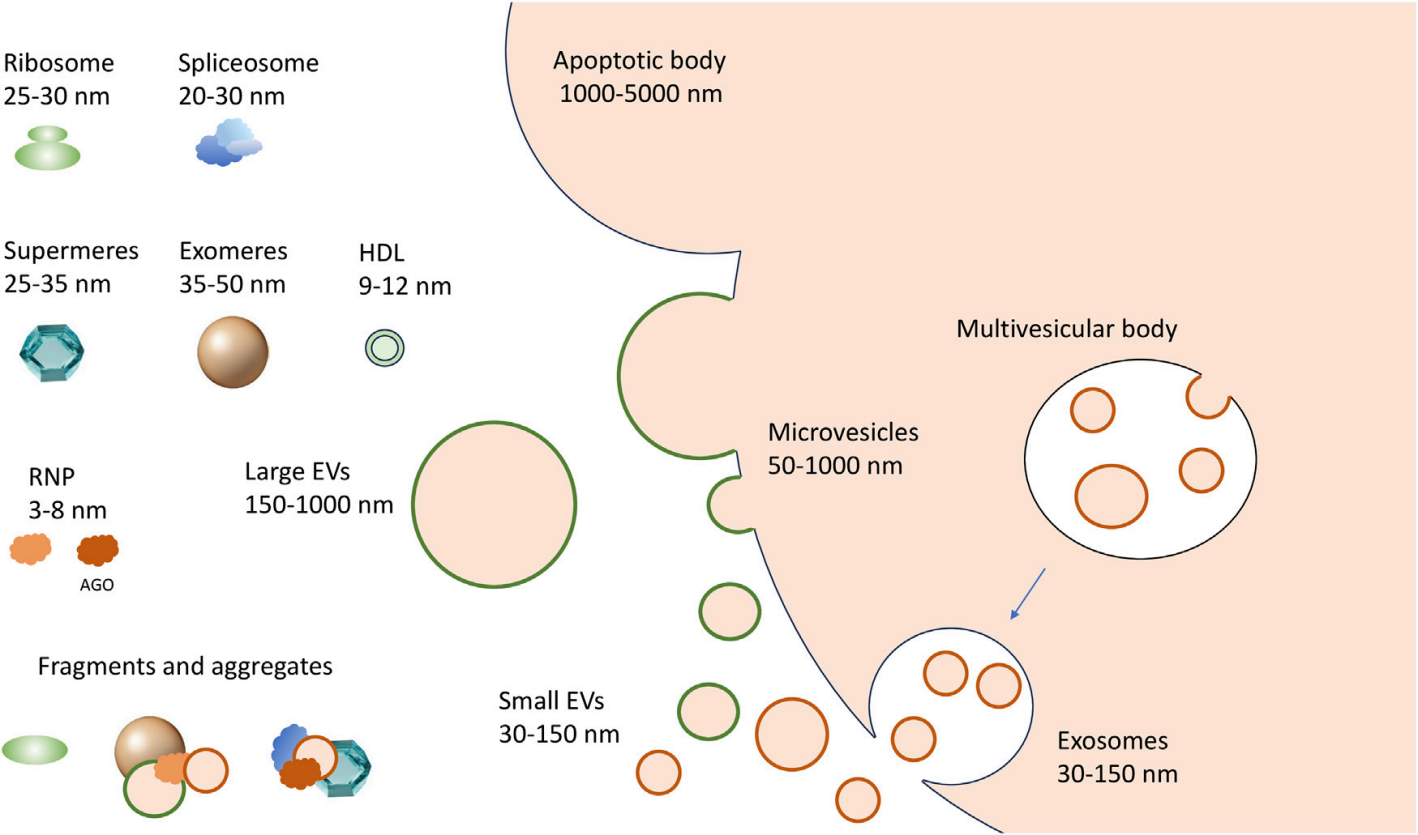
A complex spectrum of extracellular RNA carriers. Microvesicles are generated by outward budding (shedding out) from the cell membrane; the exosomes are formed by the fusion of multivesicular bodies with the cell membrane, while larger apoptotic bodies are formed during programmed cell death. The non-vesicular carriers include exomeres and supermeres. The RNP complexes that can be released from dying or damaged cells include ribosomes, spliceosomes and their fragments, and other RNA-protein complexes of different sizes, up to single proteins in complex with RNA, like AGO-miRNA complexes.

To date, experimentally confirmed exRNA carriers include extracellular vesicles (EVs), RNA binding proteins (RBPs), and large protein aggregates designated as “exomeres” and “supermeres” (Figure 1). EVs of various sizes remain the most studied exRNA carriers (Mateescu et al., 2022). Primarily, mammalian cells secrete microvesicles (MVs, also known as shedding vesicles), which are 50–1,000 nm in diameter and are formed by outward budding of the plasma membrane. The second type of EVs, the exosomes, have a diameter of 30–150 nm. They initially arise as intraluminal vesicles budding off the interior of the multivesicular bodies (MVBs) and penetrate into extracellular space upon fusion of MVBs with the plasma membrane (Figure 1) (Van Niel et al., 2022; Costa et al., 2023; Dixson et al., 2023). Cells typically secrete between 10 and 2,000 EVs of different types per cell per day (Barman et al., 2022; Garcia-Martin et al., 2022). Integrins and selectins remain well-confirmed MV markers along with membrane-associated proteins from parental cells. On the contrary, exosomes can be differentiated by the presence of tetraspanins, including CD9, CD63, and CD81 (Escola et al., 1998; Abache et al., 2007). Apoptotic bodies–the third type of membrane vesicles in the extracellular milieu–are typically between 1,000 and 5,000 nm in size and may contain cell organelles as well as nuclear fractions. Finally, the “oncosomes,” membrane vesicles exported by cancer cells, have been referred as a separate type of exRNA carriers by some authors (Di Vizio et al., 2012; Morello et al., 2013; Dixson et al., 2023). Apart from various proteins, the EV cargo typically includes complex RNA transcriptome. Thus, several online databases, including Vesiclepedia (Kalra et al., 2012), EVpedia (Kim et al., 2015), ExoCarta (Keerthikumar et al., 2016), and EV-ADD (Tsering et al., 2022) have been recently created to systematize the content of various EV types in human liquid biopsy samples.

Recently, two novel types of non-vesicular nanoparticles–exomeres and supermeres–have been isolated from the conditioned media of different cultured cells. Specifically, 35–50 nm exomeres were purified by asymmetric field-flow fractionation (AF4) (Zhang et al., 2018) or ultracentrifugation at 167,000 g (Zhang et al., 2019). Upon precipitation of exomeres, the supernatant was further subjected to high-speed ultracentrifugation at 367,000 g, resulting in sedimentation of smaller 25–35 nm particles named “supermeres” (Zhang et al., 2021). Both types of particles contained metabolic enzymes, resident cytoplasmic proteins, and significant amounts of short RNA. However, it remains unanswered if exomeres and supermeres are to any extent different from the aggregates of various RNPs which are also abundant in the extracellular environment (Tosar et al., 2022). Finally, certain miRNAs have been found in isolates of lipoprotein particles (HDL, VLDL and LDL) (Vickers et al., 2011; Florijn et al., 2019; Clément et al., 2021), but their association with VLDL and LDL remains controversial (Srinivasan et al., 2019; Rossi-Herring et al., 2023).

To understand the exact role of an exRNA carrier in cell-cell communication and to explore its potential as a disease biomarker, it is crucial to characterize the associated transcriptomes. However, the latter task has proven to be unexpectedly challenging. Primarily, the size of different EV types can be identical, while protein markers specific for each EV type have not been yet established, and apparently may not exist at all (Van Niel et al., 2022; Hendrix et al., 2023). Because only a mixed population of EVs can be obtained in any experimental setting described so far, the terms “large EV” (lEV) and “small EV” (sEV) are now increasingly used instead of “microvesicle” and “exosome,” respectively (Figure 1) (Mathieu et al., 2019). Secondly, certain non-vesicular particles and their aggregates have sizes and densities identical to EVs (Mathieu et al., 2019). Therefore, none of the existing bulk isolation methods secures complete separation of extracellular RNA carriers. In addition, a lack of standardization in applying particular approaches can result in significant discrepancies between the results obtained by different research groups. Combinations of several methods usually give significantly better separation but can be too labor-intensive and require large sample volumes. Thus, the development of more accessible and efficient techniques for separating exRNA carriers, as well as their standardization accepted by the EV community, remains of paramount importance (Théry et al., 2018; Hendrix et al., 2023).

In the following parts of the manuscript, we discuss the reported transcriptomes of various exRNA carriers and key points for their characterization, highlighting current trends, advances and challenges in the field.

## 2 Transcriptome of extracellular vesicles

To date, hundreds of original research reports have addressed transcriptomes of various biological fluids and individual exRNA carriers. As a result, several databases accumulating information on exRNA profiles in whole biological fluids and individual types of carriers have been created. For instance, the exRNA Atlas, established by the Extracellular RNA Communication Consortium, is the most extensive database predominantly featuring small RNA-seq and RT-qPCR data, encompassing both whole biofluids and EV-specific datasets (Murillo et al., 2019). The EVAtlas includes small RNA-seq data on EV fractions only (Liu et al., 2022), while exoRBase is focused on long RNA content in EV (Lai et al., 2022). Overall, exRNA Atlas data contain miRNAs (19.1%), mRNA fragments (13.8%), tRNAs (10.1%), piRNAs (0.8%) and other genome sequences (45.9%) (rRNAs are excluded on preprocessing stage). According to EVAtlas, EVs from different biological fluids and conditioned media include rRNA fragments (38.12%), miRNAs (26.94%), tRNAs (19.19%), Y RNAs (12.59%), piRNAs (1.51%), snRNAs (1.05%), and snoRNAs (0.6%).

However, the results obtained by different laboratories are highly heterogeneous. Thus, analysis of 2,756 small RNA-seq datasets from 83 studies of EV transcriptomes showed that the distribution of different RNA biotypes in EVs was poorly coherent between reports even for the same biofluid (Wang et al., 2023a). On average, the major RNA fractions in EVs isolated from blood plasma were miRNAs (39.6%), Y RNAs (15.9%), rRNA fragments (10.5%) and tRNAs (3.2%), while snRNA, sno/scaRNA, tRNA, and other RNAs accounted for less than 1%. As expected, the distribution of different exRNA biotypes strongly depended on the EVs isolation method. Notably, according to most reports, the ratio of miRNAs found in EVs was significantly lower than in cells, while tRNA and rRNA proportions were higher (Wang et al., 2023a).

Apart from short RNAs, EVs isolates contain long RNA molecules detected by 3rd-generation sequencing technologies (Padilla et al., 2023). Specifically, EVs have been shown to contain considerable amounts of intact RNA molecules (Rodosthenous et al., 2020; O’Grady et al., 2022), with full-length mRNAs enriched in lEVs (Padilla et al., 2023). mRNAs are represented mainly by the 3′UTRs (Wei et al., 2017). A total of 19,643 mRNAs and 15,646 lncRNAs, as well as pseudogenes and circRNAs were detected by long RNA sequencing in EVs according to the current release of exoRBase (Lai et al., 2022). Full-length tRNAs and YRNAs were also identified in EVs (Shurtleff et al., 2017).

Unlike sEVs, the lEVs transcriptome composition resembles that of parent cells and contains many full-length RNA molecules, including intact rRNA (Jeppesen et al., 2019). However, the reported sEVs and lEVs transcriptomes are highly heterogeneous and poorly reproducible.

Considerable variations in exRNA profiles obtained by different studies, along with limitations of current methodological approaches, facilitated the development of the so-called computational deconvolution technique, which allows to identify the ratio of different types of exRNA carriers in a given sample based on RNA-seq data from whole biological fluids and “training samples” corresponding to individual kinds of exRNA carriers (Murillo et al., 2019; Srinivasan et al., 2019). However, the precision of the method mentioned above highly depends on the quality of the original training samples, which ideally should be obtained from the cleanest possible media with well-documented identities. In their recent report, LaPlante et al. (2023) proposed an integrative analysis of eCLIP data, which implied the identification of 150 RBPs binding sites in more than 6,000 human EV samples. Specifically, 34 RBPs were indeed detected in blood plasma and conditioned media. Remarkably, the deconvolution method described above enabled attributing RBPs to certain exRNA carriers (LaPlante et al., 2023).

The overall amount of RNA associated with a given exRNA carrier is poorly understood and highly debated. Thus, several authors reported a very low proportion of EV RNA relative to the entire exRNA pool. Furthermore, blood plasma EVs isolates pretreated with protease and RNase (along with SEC-isolated EVs) had very marginal RNA content compared to that in the original plasma (Galvanin et al., 2019). Surprisingly, two research groups reported that, on average, less than one miRNA molecule is associated with sEV (Chevillet et al., 2014; Wei et al., 2017). Recently, RNA derived from gut microbiota and viruses was detected in plasma EV (Galvanin et al., 2019; Wang et al., 2023b). Although EVs with bacterial LPS were indeed found in blood plasma (Tulkens et al., 2020), great care is needed in exogenous EV RNA studies because when RNA concentrations in sequencing preparations are very low, the contribution of contaminating RNAs, which are found in both water, reagents, and columns for RNA isolation, becomes significant (Heintz-Buschart et al., 2018). Therefore, to confirm the conclusions of works such as the ones described above, it is necessary to perform control sequencing of at least water and, preferably, of washes from RNA extraction columns, if they are used, as well as the main reagents.

Nevertheless, cell-cell communication through EV-associated RNA has been hypothesized to occur via subpopulations of EVs with high RNA content, including miRNAs, and these EVs were recently found (Lee et al., 2019a; Barman et al., 2022), suggesting them as promising candidates for intercellular signaling.

## 3 Transcriptome of non-vesicular exRNA carriers

### 3.1 Non-vesicular exRNA carriers in sEV fraction

Until recently, differential ultracentrifugation at 100,000 g followed by precipitation remained a gold standard for sEV isolation (Théry et al., 2006). However, careful analysis of sEV pellets in iodixanol gradients revealed the presence of a distinct non-vesicular fraction (NVF) enriched with metabolic enzymes (GAPDH, PKM, ENO1), cytoplasmic proteins (HSP90, tubulins, ribosome proteins and translation factors), histones, and intact vault particles. In addition, all cellular RNA classes have been detected in NVF by small RNA sequencing. Interestingly, NVF RNA profiles differed significantly from both intracellular and sEV ones. Specifically, NVF was highly enriched in vault RNA, while most overrepresented miRNAs were associated with NVF and not sEVs. Furthermore, NVF fraction contained almost all AGO proteins. Long RNA sequencing revealed no significant differences between NVF and sEV exRNA profiles, with more than 40% of all reads attributed to mRNAs (Jeppesen et al., 2019).

### 3.2 Transcriptome of exomeres and supermeres

While both exomeres and supermeres contain RNA, the latter’s distribution was very uneven. Specifically, only 10% RNA was found in the exomeres, whereas the supermeres fraction contained ∼65%, and the remaining ∼25% RNA was associated with sEV-NVF (precipitate after ultracentrifugation at 167,000 g) (Zhang et al., 2021). However, the RNA content of lEV and supermeres supernatants was not reported, and thus the full spectrum of exRNA remained unaddressed. According to small RNA sequencing data, all major small RNA biotypes have been found in exomeres and supermeres, with miRNA reads significantly prevailed (more than 60% of all reads in the supermeres and 79% in the exomeres). Furthermore, both types of particles were enriched in AGO proteins. Interestingly, expression patterns of miRNAs in exomeres and supermeres were similar but differed significantly from sEV-NVF and cells (Zhang et al., 2018; Jeppesen et al., 2019; Zhang et al., 2019; Zhang et al., 2021).

The mechanisms of biogenesis and the exact biological role of exomeres and supermeres remain to be addressed. Their protein composition resembles that of NVF-fraction, so the question arises whether and to what extent aggregates of exomeres and supermeres form NVFs. Whether exomeres and supermeres represent discrete particles with unique protein sets and distinct biogenesis remains unknown. It is feasible, however, that both exomeres and supermeres are, in fact, continuous series of cellular RNPs of different sizes. The point is that the extracellular environment contains ribosomes and their fragments, as well as fragments of spliceosomes, and both ribosome and spliceosome proteins were detected in supermeres and exomeres (Tosar et al., 2020; Zhang et al., 2021; Tosar et al., 2022). The size of ribosomes and spliceosomes corresponds to the size of supermeres (Figure 1). Therefore, it is feasible that both exomeres and supermeres represent extracellular RNP fractions consisting of a set of intracellular RNP and protein complexes of different sizes and their fragments (Tosar et al., 2022). This, however, does not exclude the possibility of the existence of secreted particles of a certain composition in these fractions (i.e., *bona fide* exomeres and supermeres) (Jeppesen et al., 2022). Another question is whether the fractions of these particles are free of vesicles. Thus, ultracentrifugation at 200,000 g shows evidence of the presence of very small vesicles in the precipitate corresponding to the exomeres (Lee et al., 2019b). However, whatever non-vesicular exRNA carriers consist of, they are likely to serve as a new page in the story of cell-cell communication: it has already been shown that exomeres can deliver functionally active protein cargo to recipient cells (Zhang et al., 2019). And since it is the non-vesicular exRNA carriers where the bulk of exRNAs, including miRNAs as well as AGO proteins, are concentrated, they may prove to be an important actor in this story. In this regard, it is interesting to note the recent demonstration that non-vesicular miRNA-AGO complexes in the extracellular fluids are indeed functional and capable of efficient silencing (Geekiyanage et al., 2020).

## 4 Challenges in the exRNA profiling

The heterogeneity of the results obtained by different research groups could stem from methods used for RNA conversion into cDNA. Specifically, widely used RNA-seq library preparation approaches produce different biases for both long (Rodosthenous et al., 2020) and small RNA sequencing (Srinivasan et al., 2019). For instance, a comparison of six different cDNA library preparation methods for long RNA-seq demonstrated drastic variation in mapping efficacy and proportions of different RNA classes detected (Rodosthenous et al., 2020). It should be noted that the vast majority of previous reports on small RNA sequencing of exRNA used standard cDNA library preparation protocols based on the ligation of adapters to both termini of small RNA. By default, such ligation reaction requires the presence of phosphate at the 5′-end of the RNA and hydroxyl at the 3′-end. The ligation-based methods were originally tailored for capturing intracellular miRNAs that are 5′-phosphorylated, and until recently, the biases associated with standard ligation-based commercial kits have not been considered. However, extracellular RNAs are exposed to RNases and as a result are converted to 5′-hydroxyl and 3′-(cyclo) phosphate entities (Sorrentino, 2010; Giraldez et al., 2019; Nechooshtan et al., 2020). Because ssRNA adapters cannot be ligated to such RNAs, the latter will not be included into the final library and sequenced without prior end-repair. Therefore, the distortions of most currently reported exRNA profiles can be very significant. Such distortions can be avoided by prior treatment of samples with T4 polynucleotide kinase (T4 PNK), which phosphorylates the 5′-ends of RNA and removes phosphates/cyclophosphates from the 3′-termini. Thus, deep sequencing of T4 PNK-treated extracellular small RNAs demonstrated that in lEVs, sEVs, NVF and in whole plasma libraries ∼80–90% were rRNA fragments (Wei et al., 2017; Akat et al., 2019; Giraldez et al., 2019; Solaguren-Beascoa et al., 2023). In rRNA-depleted libraries, the percentage of reads corresponding to mRNA and lncRNA fragments was increased up to 10-fold (Giraldez et al., 2019), while lEVs and sEVs contained ∼25% repetitive sequences, and NVF contained ∼45% tRNAs. Thus, most exRNAs are degradation products of various long RNAs and tRNAs, with a very small proportion of miRNAs and other small RNA types.

Importantly, exRNA profiling has several previously underestimated crucial methodological issues that should ideally be addressed in every experimental setting. To facilitate this process, we offer a guide containing the key experiments required for the correct profiling of exRNA carriers (Table 1). Further progress in characterizing the exRNA population is expected upon application of recently emerged RNA-seq library preparation methods such as D-Plex small RNA-seq kit (Diagenode), SMARTer smRNA-seq kit (Takara) and BioLiqX small RNA-seq kit (Heidelberg Biolabs), which enable unbiased incorporation of short RNA fragments with modified nucleotides. Wide application of novel RNA sequencing methods can confirm exRNA transcriptomes of exomeres, supermeres and whole biological fluids.

**TABLE 1:** Key experiments in exRNA profiling.

Carrier	Action	Effect	Suitable for methods
EV	Treatment of EVs with proteases and RNases	Removes co-purified non-vesicular RNPs	All except purification in density gradients and immunopurification
Non-EV	De-phosphorylation of RNA with T4 PNK before generating a cDNA library	The cDNA library will include total RNA from the non-EV fraction	All
All	RNA-seq of cell medium prior to cell culture	Accounting for background levels of non-related exRNAs	All
All	RNA-seq of water used for RNA elution and/or column washout	The background RNA will be taken into account and will not be mistaken for exRNA	All
All	RNA-seq of reagents used (buffers, column washes) and selection of the best suppliers	Selection of the purest reagents with minimal background RNA	All

## 5 Conclusion and future directions

The experimentally confirmed exRNA carriers list includes lEVs, several types of sEVs, single RBPs, exomeres and supermeres. Biofluids apparently contain a set of membrane vesicles of continuous size range–up to the minimum physically possible, as well as a set of proteins and RNP aggregates ranging up to individual proteins. Furthermore, the concentration of a given exRNA carrier could be labile and prone to change under different physiologic and pathologic conditions. Apart from that, methodological obstacles and biological diversity explain the marked variation in the transcriptomic content of exRNA carriers obtained by different groups. Thus, the introduction of unified experimental standards for exRNA purification and detection is of utmost importance. Further systematic profiling of individual subfractions, such as immunopurified EVs and RNPs, may enable the compilation of a comprehensive extracellular transcriptome map and the creation of relevant training samples for computational deconvolution of the exRNA transcriptome in biofluids.
